# Supplementary material for: Discovery and application of insertion-deletion (INDEL) polymorphisms for QTL mapping of early life-history traits in Atlantic salmon
Source: BMC Genomics. 2010 Mar 8;11:156. doi: 10.1186/1471-2164-11-156 (PMC2838853; doi:10.1186/1471-2164-11-156)
Supplement: Additional file 2 — Information on developed 76 locus single-run INDEL panel in Atlantic salmon. Information on fluorescence labeling, primer concentrations, PCR pooling and links to alignments, INDEL motifs and GENESCAN (Burge and Karlin 1997) predictions of genes/exons are available in html format. [file 1471-2164-11-156-S2.ZIP › Additionalfile2/table5397.html]

Contig Alignment

```
Base	A B C D E F G H I J K L M N O P Q R S T U V W X Y   
1	. . G . . G . . . . . . . . . . . . . . . . . . .    
2	. . A . . A . . . . . . . . . . . . . . . . . . .    
3	. . G . . G . . . . . . . . . . . . . . . . . . .    
4	. . A . . A . . . . . . . . . . . . . . . . . . .    
5	. . C . . C . . . . . . . . . . . . . . . . . . .    
6	. . T . . T . . . . . . . . . . . . . . . . . . .    
7	. . T . . T . . . . . . . . . . . . . . . . . . .    
8	. C C . . C C . . . . . . . . . . . . . . . . . .    
9	. A A . . A A . . . . . . . . . . . . . . . . . .    
10	. C C . . C C . . . . . . . . . . . . . . . . . .    
11	. A A . . A A . . . . . . . . . . . . . . . . . .    
12	. A A . . A A . . . . . . . . . . . . . . . . . .    
13	. C C . . C C . . . . . . . . . . . . . . . . . .    
14	A A A A . A A . . . . . . . . . . . . . . . . . .    
15	A A A A . A A . . . . . . . . . . . . . . . . . .    
16	A A A A . A A . . . . . . . . . . . . . . . . . .    
17	G G G G . G G . . . . . . . . . . . . . . . . . .    
18	C C C C . C C . . . . . . . . . . . . . . . . . .    
19	T T T T . T T . . . . . . . . . . . . . . . . . .    
20	C C C C . C C . . . . . . . . . . . . . . . . . .    
21	C C C C . C C . . . . . . . . . . . . . . . . . .    
22	A A A A . A A . . . . . . . . . . . . . . . . . .    
23	T T T T . T T . . . . . . . . . . . . . . . . . .    
24	G G G G . G G . . . . . . . . . . . . . . . . . .    
25	T T T T . T T . . . . . . . . . . . . . . . . . .    
26	T T T T . T T . . . . . . . . . . . . . . . . . .    
27	T T T T . T T . . . . . . . . . . . . . . . . . .    
28	T T T T . T T . . . . . . . . . . . . . . . . . .    
29	A A A A . A A . . . . . . . . . . . . . . . . . .    
30	T T T T . T T . . . . . . . . . . . . . . . . . .    
31	T T T T . T T . . . . . . . . . . . . . . . . . .    
32	A A A A . A A . . . . . . . . . . . . . . . . . .    
33	A A A A . A A . . . . . . . . . . . . . . . . . .    
34	A A A A . A A . . . . . . . . . . . . . . . . . .    
35	A A A A . A A . . . . . . . . . . . . . . . . . .    
36	C C C C . C C . . . . . . . . . . . . . . . . . .    
37	A A A A . A A . . . . . . . . . . . . . . . . . .    
38	A A A A . A A . . . . . . . . . . . . . . . . . .    
39	G G G G . G G . . . . . . . . . . . . . . . . . .    
40	A A A A . A A . . . . . . . . . . . . . . . . . .    
41	- A A A . A A . . . . . . . . . . . . . . . . . .    
42	- A A A A A A . . . A . . . . . . . . . . . . . .    
43	- G G G G G G . G G G . . G . . . . . . . . . . .    
44	- A A A A A A . A A A . . A . . . . . . . . . . .    
45	C C C C C C C . C C C . . C . . . . . . . . . . .    
46	A A A A A A A . A A A . . A . . . . . . . . . . .    
47	G G G G G G G . G G G . . G . . . . . . . . . . .    
48	A A A A A A A . A A A . . A . . . . . . . . . . .    
49	A A A A A A A . A A A . . A . . . . . . . . . . .    
50	T T T T T T T . T T T . . T . . . . . . . . . . .    
51	T T T T T T T . T T T . . T . . . . . . . . . . .    
52	A A A A A A A . A A A . . A . . . . . . . . . . .    
53	C C C C C C C . C C C . . C . . . . . . . . . . .    
54	A A A A A A A . A A A . . A . . . . . . . . . . .    
55	C C C C C C C . C C C . . C C . . . . . . . . . .    
56	A A A A A A A . A A A . . A A . . . . . . . . . .    
57	C C C C C C C . C C C . . C C . . . . . . . . . .    
58	A A A A A A A . A A A . . A A . . . . . . . . . .    
59	A A A A A A A . A A A . . A A . . . . . . . . . .    
60	C C C C C C C . C C C . . C C . . . . . . . . . .    
61	T T T T T T T . T T T . . T T . . . . . . . . . .    
62	A A A A A A A . A A A . . A A . . . . . . . . . .    
63	G G G G G G G . G G G . . G G . . . . . . . . . .    
64	G G G G G G G . G G G . . G G . . . . . . . . . .    
65	C C C C C C C . C C C . . C C . . . . . . . . . .    
66	A A A A A A A . A A A . . A A . . . . . . . . . .    
67	C C C C C C C . C C C . . C C . . . . . . . . . .    
68	A A A A A A A . A A A . . A A . . . . . . . . . .    
69	A A A A A A A . A A A . . A A . . . . . . . . . .    
70	A A A A A A A . A A A . . A A . . . . . . . . . .    
71	T T T T T T T . T T T . . T T . . . . . . . . . .    
72	G G G G G G G . G G G . . G G . . . . . . . . . .    
73	T T T T T T T . T T T . . T T . . . . . . . . . .    
74	A A A A A A A . A A A . . A A . . . . . . . . . .    
75	C C C C C C C . C C C . . C C . . . . . . . . . .    
76	A A A A A A A . A A A . . A A . . . . . . . . . .    
77	A A A A A A A . A A A . . A A . . . . . . . . . .    
78	T T T T T T T . T T T . . T T . . . . . . . . . .    
79	T T T T T T T T T T T . . T T . . . . . . . . . .    
80	G G G G G G G G G G G . . G G . . . . . . . . . .    
81	A A A A A A A A A A A . . A A . . . . . . . . . .    
82	A A A A A A A A A A A . . A A . . . . . . . . . .    
83	A A A A A A A A A A A . . A A . . . . . . . . . .    
84	A A A A A A A A A A A . . A A . . . . . . . . . .    
85	A A A A A A A A A A A . . A A . . . . . . . . . .    
86	G G G G G G G G G G G . . G G . . . . . . . . . .    
87	T T T T T T T T T T T . . T T . . . . . . . . . .    
88	T T T T T T T T T T T . . T T . . . . . . . . . .    
89	A A A A A A A A A A A . . A A . . . . . . . . . .    
90	A A A A A A A A A A A . . A A . . . . . . . . . .    
91	A A A A A A A A A A A . . A A . . . . . . . . . .    
92	A A A A A A A A A A A . . A A . . . . . . . . . .    
93	T T T T T T T T T T T . . T T . . . . . . . . . .    
94	A A A A A A A A A A A . . A A . . . . . . . . . .    
95	A A A A A A A A A A A . . A A . . . . . . . . . .    
96	A A A A A A A A A A A . . A A . . . . . . . . . .    
97	A A A A A A A A A A A . . A A . . . . . . . . . .    
98	C C C C C C C C C C C . . C C . . . . . . . . . .    
99	C C C C C C C C C C C . . C C . . . . . . . . . .    
100	T T T T T T T T T T T . . T T . . . . . . . . . .    
101	T T T T T T T T T T T . . T T . . . . . . . . . .    
102	A A A A A A A A A A A . . A A . . . . . . . . . .    
103	T T T T T T T T T T T . . T T . . . . . . . . . .    
104	C C C C C C C C C C C . . C C . . . . . . . . . .    
105	T T T T T T T T T T T . . T T . . . . . . . . . .    
106	A A A A A A A A A A A . . A A . . . . . . . . . .    
107	C C C C C C C C C C C . . C C . . . . . . . . . .    
108	T T T T T T T T T T T . . T T . . . . . . . . . .    
109	T T T T T T T T T T T . . T T . . . . . . . . . .    
110	T T T T T T T T T T T . . T T . . . . . . . . . .    
111	C C C C C C C C C C C . . C C . . . . . . . . . .    
112	T T T T T T T T T T T . . T T . . . . . . . . . .    
113	A A A A A A A A A A A . . A A . . . . . . . . . .    
114	A A A A A A A A A A A . . A A . . . . . . . . . .    
115	T T T T T T T T T T T . . T T . . . . . . . . . .    
116	T T T T T T T T T T T . . T T . . . . . . . . . .    
117	C C C C C C C C C C C . . C C . C . . . . . . . .    
118	C C C C C C C C C C C . . C C . C . . . . . . . .    
119	C C C C C C C C C C C . . C C . C . . . . . . . .    
120	T T T T T T T T T T T . . T T . T . . . . . . . .    
121	T T T T T T T T T T T . . T T . T . . . . . . . .    
122	T T T T T T T T T T T . . T T . T . . . . . . . .    
123	G G G G G G G G G G G . . G G . G . . . . . . . .    
124	G G G G G G G G G G G . . G G . G . . . . . . . .    
125	T T T T T T T T T T T . . T T . T . . . . . . . .    
126	C C C C C C C C C C C . . C C . C . . . . . . . .    
127	C C C C C C C C C C C . . C C . C . . . . . . . .    
128	A A A A A A A A A A A . . A A . A . . . . . . . .    
129	T T T T T T T T T T T . . T T . T . . . . . . . .    
130	T T T T T T T T T T T . . T T . T . . . . . . . .    
131	T T T T T T T T T T T . . T T . T . . . . . . . .    
132	A A A A A A A A A A A . . A A . A . . . . . . . .    
133	C C C C C C C C C C C . . C C . C . . . . . . . .    
134	A A A A A A A A A A A . . A A . A . . . . . . . .    
135	C C C C C C C C C C C . . C C . C . . . . . . . .    
136	A A A A A A A A A A A . . A A . A . . . . . . . .    
137	T T T T T T T T T T T . . T T . T . . . . . . . .    
138	G G G G G G G G G G G . . G G . G . . . . . . . .    
139	G G G G G G G G G G G . . G G . G . . . . . . . .    
140	T G T G T T G T T T G . . T T . T . . . . . . . .    
141	G G G G G G G G G G G . . G G . G . . . . . . . .    
142	G G G G G G G G G G G . . G G . G . . . . . . . .    
143	T G T T T T T T T T T . . T T . T . . . . . . . .    
144	G G G G G G G G G G G . . G G . G . . . . . . . .    
145	C C C C C C C C C C C . . C C . C . . . . . . . .    
146	T T T T T T T T T T T . . T T . T . . . . . . . .    
147	G G G G G G G G G G G . . G G . G . . . . . . . .    
148	T T T T T T T T T T T . . T T . T . . . . . . . .    
149	G G G G G G G G G G G . . G G . G . . . . . . . .    
150	C C C C C C C C C C C . . C C . C . . . . . . . .    
151	A A A A A A A A A A A . . A A . A . . . . . . . .    
152	T T T T T T T T T T T . . T T . T . . . . . . . .    
153	C C C C C C C C C C C . . C C . C . . . . . . . .    
154	T T T T T T T T T T T . . T T . T . . . . . . . .    
155	T T T T T T T T T T T . . T T . T . . . . . . . .    
156	T T T T T T T T T T T . . T T . T . . . . . . . .    
157	A A A A A A A A A A A . . A A . A . . . . . . . .    
158	A A A A A A A A A A A . . A A . A . . . . . . . .    
159	T T T T T T T T T T T . . T T . T . . . . . . . .    
160	A A A A A A A A A A A . . A A . A . . . . . . . .    
161	A A A A A A A A A A A . . A A . A . . . . . . . .    
162	A A A A A A A A A A A . . A A . A . . . . . . . .    
163	G G G G G G G G G G G . . G G . G . . . . . . . .    
164	G G G G G G G G G G G . . G G . G . . . . . . . .    
165	T T T T T T T T T T T . . T T . T . . . . . . . .    
166	G G G G G G G G G G G . . G G . G . . . . . . . .    
167	A A A A A A A A A A A . . A A . A . . . . . . . .    
168	A A A A A A A A A A A . . A A . A . . . . . . . .    
169	G G G G G G G G G G G . . G G . G . . . . . . . .    
170	T T T T T T T T T T T . . T T . T . . . . . . . .    
171	G G G G G G G G G G G . . G G . G . . . . . . . .    
172	A A A A A A A A A A A . . A A . A . . . . . . . .    
173	C C C C C C C C C C C . . C C . C . . . . . . . .    
174	T T T T T T T T T T T . . T T . T . . . . . . . .    
175	G G G G G G G G G G G . . G G . G . . . . . . . .    
176	T T T T T T T T T T T . . T T . T . . . . . . . .    
177	A A A A A A A A A A A . . A A . A . . . . . . . .    
178	A A A A A A A A A A A . . A A . A . . . . . . . .    
179	A A A A A A A A A A A . . A A . A . . . . . . . .    
180	T T T T T T T T T T T . . T T . T . . . . . . . .    
181	G G G G G G G G G G G . . G G . G . . . . . . . .    
182	G G G G G G G G G G G . . G G . G . . . . . . . .    
183	T T T T T T T T T T T . . T T . T . . . . . . . .    
184	A A A A A A A A A A A . . A A . A . . . . . . . .    
185	T T T T T T T T T T T . . T T . T . . . . . . . .    
186	G G G G G G G G G G G . . G G . G . . . . . . . .    
187	A A A A A A A A A A A . . A A . A . . . . . . . .    
188	G G G G G G G G G G G . . G G . G . . . . . . . .    
189	T T T T T T T T T T T . . T T . T . . T . . . . .    
190	C C C C C C C C C C C . . C C . C . . C . . . . .    
191	A A A A A A A A A A A . . A A . A . . A . . . . .    
192	G A G G A G A G G G G . . G G . G . . G . . . . .    
193	A A A A A A A A A A A . . A A . A . . A . . . . .    
194	G G G G G G G G G G G . . G G . G . . G . . . . .    
195	T T T T T T T T T T T . . T T . T . . T . . . . .    
196	T T T T T T T T T T T . . T T . T . . T . . . . .    
197	A A A A A A A A A A A . . A A . A . . A . . . . .    
198	A A A A A A A A A A A . . A A . A . . A . . . . .    
199	C C C C C C C C C C C . . C C . C . . C . . . . .    
200	A A A A A A A A A A A . . A A . A . . A . . . . .    
201	T T T T T T T T T T T . . T T . T . . T . . . . .    
202	T T T T T T T T T T T . . T T . T . . T . . . . .    
203	G G G G G G G G G G G . . G G . G . . G . . . . .    
204	A A A A A A A A A A A . . A A . A . . A . . . . .    
205	A A A A A A A A A A A . . A A . A . . A . . . . .    
206	A A A A A A A A A A A . . A A . A . . A . . . . .    
207	A A A A A A A A A A A . . A A . A . . A . . . . .    
208	A A A A A A A A A A A . . A A . A . . A . . . . .    
209	A A A A A A A A A A A . . A A . A . . A . . . . .    
210	T T T T T T T T T T T . . T T . T . . T . . . . .    
211	. G G G G G G G G G G . . G G . G . . G . . . . .    
212	. C C C C C C C C C C . . C C . C . . C . . . . .    
213	. T T T T T T T T T T . . T T . T . . T . . . . .    
214	. G G G G G G G G G G . . G G . G . . G . . . . .    
215	. A A A A A A A A A A . . A A . A . . A . . . . .    
216	. C C C C C C C C C C . . C C . C . . C . . . . .    
217	. T T T T T T T T T T . . T T . T . . T . . . . .    
218	. T T T T T T T T T T . . T T . T . . T . . . . .    
219	. T T T T T T T T T T . . T T . T . . T . . . . .    
220	. G G G G G G G G G G . . G G . G . . G . . . . .    
221	. A A A A A A A A A A . . A A . A . . A . . . . .    
222	. C C C C C C C C C C . . C C . C . . C . . . . .    
223	. C C C C C C C C C C . . C C . C . . C . . . . .    
224	. A A A A A A A A A A . . A A . A . . A . . . . .    
225	. G G G G G G G G G G . . G G . G . . G . . . . .    
226	. A A A A A A A A A A . . A A . A . . A . . . . .    
227	. A A A A A A A A A A . . A A . A . . A . . . . .    
228	. G G G G G G G G G G . . G G . G . . G . . . . .    
229	. C C C C C C C C C C . . C C . C . . C . . . . .    
230	. A A A A A A A A A A . . A A . A . . A . . . . .    
231	. C C C C C C C C C C . . C C . C . . C . . . . .    
232	. T T T T T T T T T T . . T T . T . . T . . . . .    
233	. T T T T T T T T T T . . T T . T . . T . . . . .    
234	. T T T T T T T T T T . . T T . T . . T . . . . .    
235	. G G G G G G G G G G . . G G . G . . G . . . . .    
236	. T T T T T T T T T T . . T T . T . . T . . . . .    
237	. A A A A A A A A A A . . A A . A . . A . . . . .    
238	. C C C C C C C C C C C . C C . C . . C . . . . .    
239	. T T T T T T T T T T T T T T . T . . T . . . . .    
240	. A A A A A A A A A A A A A A . A . . A . . . . .    
241	. C C C C C C C C C C C C C C . C . . C . . . . .    
242	. T T T T T T T T T T T T T T . T . . T . . . . .    
243	. G G G G G G G G G G G G G G . G . . G . . . . .    
244	. G G G G G G G G G G G G G G . G . . G . . . . .    
245	. G G G G G G G G G G G G G G . G . . G . . . . .    
246	. G G G G G G G G G G G G G G . G . . G . . . . .    
247	. T T T T T T T T T T T T T T . T . . T . . . . .    
248	. T T T T T T T T T T T T T T . T . . T . . . . .    
249	. T T T T T T T T T T T T T T . T . . T . . . . .    
250	. C C C C C C C C C C C C C C . C . . C . . . . .    
251	. C C C C C C C C C C C C C C . C . . C . . . . .    
252	. T T T T T T T T T T T T T T . T . . T . . . . .    
253	. C C C C C C C C C C C C C C . C . . C . . . . .    
254	. T T T T T T T T T T T T T T . T . . T . . . . .    
255	. A A A A A A A A A A A A A A . A . . A . . . . .    
256	. C C C C C C C C C C C C C C . C . . C . . . . .    
257	. A A A A A A A A A A A A A A . A . . A . . . . .    
258	. A A A A A A A A A A A A A A . A . . A . . . . .    
259	. G G G G G G G G G G G G G G . G . . G . . . . .    
260	. C C C C C C C C C C C C C C . C . . C . . . . .    
261	. T T T T T T T T T T T T T T . T . . T . . . . .    
262	. C C C C C C C C C C C C C C . C . . C . . . . .    
263	. T T T T T T T T T T T T T T . T . . T . . . . .    
264	. G G G G G G G G G G G G G G . G . . G . . . . .    
265	. A A A A A A A A A A A A A A . A . . A . . . . .    
266	. G G G G G G G G G G G G G G . G . . G . . . . .    
267	. G G G G G G G G G G G G G G . G . . G . . . . .    
268	. A A A A A A A A A A A A A A . A . . A . . . . .    
269	. A A A A A A A A A A A A A A . A . . A . . . . .    
270	. G G G G G G G G G G G G G G . G . . G . . . . .    
271	. T T T T T T T T T T T T T T . T . . T . . . . .    
272	. C C C C C C C C C C C C C C . C . . C . . . . .    
273	. C C C C C C C C C C C C C C . C . . C . . . . .    
274	. A A A A A A A A A A A A A A . A . . A . . . . .    
275	. C C C C C C C C C C C C C C . C . . C . . . . .    
276	. A A A A A A A A A A A A A A . A . . A . . . . .    
277	. A A A A A A A A A A A A A A . A . . A . . . . .    
278	. A A A A A A A A A A A A A A . A . . A . . . . .    
279	. A A A A A A A A A A A A A A . A . . A . . . . .    
280	. C C C C C C C C C C C C C C . C . . C . . . . .    
281	. T T T T T T T T T T T T T T . T . . T . . . . .    
282	. A A A A A A A A A A A A A A . A . . A . . . . .    
283	. T T T T T T T T T T T T T T . T . . T . . . . .    
284	. A A A A A A A A A A A A A A . A . . A . . . . .    
285	. A A A A A A A A A A A A A A . A . . A . . . . .    
286	. A A A A A A A A A A A A A A . A . . A . . . . .    
287	. T T T T T T T T T T T T T T . T . . T . . . . .    
288	. A A A A A A A A A A A A A A . A . . A . . . . .    
289	. - - - - - - - - - - N - - - . - . . - . . . . .    
290	. A A A A A A A A A A A A A A . A . . A . . . . .    
291	. C C C C C C C C C C C C C C . C . . C . . . . .    
292	. T T T T T T T T T T T T T T . T . . T . . . . .    
293	. C C C C C C C C C C C C C C . C . . C . . . . .    
294	. T T T T T T T T T T T T T T . T . . T . . . . .    
295	. T T T T T T T T T T T T T T . T . . T . . . . .    
296	. A A A A A A A A A A A A A A . A . . A . . . . .    
297	. C C C C C C C C C C C C C C . C . . C . . . . .    
298	. C C C C C C C C C C C C C C . C . . C . . . . .    
299	. T T T T T T T T T T T T T T . T . . T . . . . .    
300	. C C C C C C C C C C C C C C . C . . C . . . . .    
301	. T T T T T T T T T T T T T T . T . . T . . . . .    
302	. C C C C C C C C C C C C C C C C . C C . . . . .    
303	. A A A A A A A A A A A A A A A A . A A . . . . .    
304	. G G G G G G G G G G G G G G G G . G G . . . . .    
305	. T T T T T T T T T T T T T T T T . T T . . . . .    
306	. T T T T T T T T T T T T T T T T . T T . . . . .    
307	. G G G G G G G G G G G G G G G G . G G . . . . .    
308	. C C C C C C C C C C C C C C C C . C C . . . . .    
309	. T T T T T T T T T T T T T T T T . T T . . . . .    
310	. T T T T T T T T T T T T T T T T . T T . . . . .    
311	. A A A A A A A A A A A A A A A A . A A . . . . .    
312	. A A A A A A A A A A A A A A A A . A A . . . . .    
313	. C C C C C C C C C C C C C C C C . C C . . . . .    
314	. A A A A A A A A A A A A A A A A . A A . . . . .    
315	. C C C C C C C C C C C C C C C C . C C . . . . .    
316	. T T T T T T T T T T T T T T T T . T T . . . . .    
317	. A A A A A A A A A A A A A A A A . A A . . . . .    
318	. A A A A A A A A A A A A A A A A . A A . . . . .    
319	. T T T T T T T T T T T T T T T T . T T . . . . .    
320	. T T T T T T T T T T T T T T T T . T T . . . . .    
321	. T T T T T T T T T T T T T T T T T T T . . . . .    
322	. G G A G G G G G G G G G G G A G G G G . . . . .    
323	. T T T T T T T T T T T T T T T T T T T . T . . .    
324	. T T T T T T T T T T T T T T T T T T T . T . . .    
325	. T T T T T T T T T T T T T T T T T T T . T . . .    
326	. T T T T T T T T T T T T T T T T T T T . T . . .    
327	. C C C C C C C C C C C C C C C C C C C . C . . .    
328	. T T T T T T T T T T T T T T T T T T T . T . . .    
329	. T T T T T T T T T T T T T T T T T T T . T T . .    
330	. A A A A A A A A A A A A A A A A A A A . A A . .    
331	. A A A A A A A A A A A A A A A A A A A . A A . .    
332	. C C C C C C C C C C C C C C C C C C C . C C . .    
333	. G G G G G G G G G G G G G G G G G G G . G G . .    
334	. C C C C C C C C C C C C C C C C C C C . C C . .    
335	. A A A A A A A A A A A A A A A A A A A . A A . .    
336	. T T T T T T T T T T T T T T T T T T T . T T . .    
337	. A A A A A A A A A A A A A A A A A A A . A A . .    
338	. T T T T T T T T T T T T T T T T T T T . T T . .    
339	. C C C C C C C C C C C C C C C C C C C . C C . .    
340	. A A A A A A A A A A A A A A A A A A A . A A . .    
341	. T T T T T T T T T T T T T T T T T T T . T T . .    
342	. A A A A A A A A A A A A A A A A A A A . A A . .    
343	. T T T T T T T T T T T T T T T T T T T . T T . .    
344	. T T T T T T T T T T T T T T T T T T T . T T . .    
345	. T T T T T T T T T T T T T T T T T T T . T T . .    
346	. A A A A A A A A A A A A A A A A A A A . A A . .    
347	. A A A A A A A A A A A A A A A A A A A . A A . .    
348	. G G G G G G G G G G G G G G G G G G G . G G . .    
349	. A A A A A A A A A A A A A A A A A A A . A A . .    
350	. C C C C C C C C C C C C C C C C C C C . C C . .    
351	. T T T T T T T T T T T T T T T T T T T . T T . .    
352	. T T T T T T T T T T T T T T T T T T T . T T . .    
353	. T T T T T T T T T T T T T T T T T T T . T T . .    
354	. T T T T T T T T T T T T T T T T T T T . T T . .    
355	. T T T T T T T T T T T T T T T T T T T . T T . .    
356	. C C C C C C C C C C C C C C C C C C C . C C . .    
357	. C C C C C C C C C C C C C C C C C C C . C C . .    
358	. T T T T T T T T T T T T T T T T T T T . T T . .    
359	. T T T T T T T T T T T T T T T T T T T . T T . .    
360	. T T T T T T T T T T T T T T T T T T T . T T . .    
361	. T T T T T T T T T T T T T T T T T T T . T T . .    
362	. A A A A A A A A A A A A A A A A A A A . A A . .    
363	. C C C C C C C C C C C C C C C C C C C . C C . .    
364	. T T T T T T T T T T T T T T T T T T T . T T . .    
365	. C C C C C C C C C C C C C C C C C C C . C C . .    
366	. A A A A A A A A A A A A A A A A A A A . A A . .    
367	. A A A A A A A A A A A A A A A A A A A . A A . .    
368	. A A A A A A A A A A A A A A A A A A A . A A . .    
369	. T T T T T T T T T T T T T T T T T T T . T T . .    
370	. C C C C C C C C C C C C C C C C C C C . C C . .    
371	. A A A A A A A A A A A A A A A A A A A . A A . .    
372	. A A A A A A A A A A A A A A A A A A A . A A . .    
373	. G G G G G G G G G G G G G G G G G G G . G G . .    
374	. A A A A A A A A A A A A A A A A A A A . A A . .    
375	. C C C C C C C C C C C C C C C C C C C . C C . .    
376	. T T T T T T T T T T T T T T T T T T T . T T . .    
377	. A A A A A A A A A A A A A A A A A A A . A A . .    
378	. G G G G G G G G G G G G G G G G G G G . G G . .    
379	. C C C C C C C C C C C C C C C C C C C . C C . .    
380	. T T T T T T T T T T T T T T T T T T T . T T . .    
381	. A A A A A A A A A A A A A A A A A A A . A A . .    
382	. A A A A A A A A A A A A A A A A A A A . A A . .    
383	. C C C C C C C C C C C C C C C C C C C . C C . .    
384	. A A A A A A A A A A A A A A A A A A A . A A . .    
385	. G G G G G G G G G G G G G G G G G G G . G G . .    
386	. C C C C C C C C C C C C C C C C C C C . C C . .    
387	. A A A A A A A A A A A A A A A A A A A . A A . .    
388	. G G G G G G G G G G G G G G G G G G G . G G . .    
389	. C C C C C C C C C C C C C C C C C C C . C C . .    
390	. A A A A A A A A A A A A A A A A A A A . A A . .    
391	. A A A A A A A A A A A A A A A A A A A . A A . .    
392	. C C C C C C C C C C C C C C C C C C C . C C . .    
393	. A A A A A A A A A A A A A A A A A A A . A A . .    
394	. C C C C C C C C C C C C C C C C C C C . C C . .    
395	. A A A A A A A A A A A A A A A A A A A . A A . .    
396	. C C C C C C C C C C C C C C C C C C C . C C . .    
397	. A A A A A A A A A A A A A A A A A A A . A A . .    
398	. C C C C C C C C C C C C C C C C C C C . C C . .    
399	. A A A A A A A A A A A A A A A A A A A . A A . .    
400	. T T T T T T T T T T T T T T T T T T T . T T . .    
401	. T T T T T T T T T T T T T T T T T T T . T T . .    
402	. T T T T T T T T T T T T T T T T T T T . T T . .    
403	. - - - T - - - - - - - - - - - - - - - . - - . .    
404	. A A A A A A A A A A A A A A A A A A A . A A . .    
405	. A A A A A A A A A A A A A A A A A A A . A A . .    
406	. C C C C C C C C C C C C C C C C C C C . C C . .    
407	. A A A A A A A A A A A A A A A A A A G . A A . .    
408	. A A A A A A A A A A A A A A A A A A A . A A . .    
409	. A A A A A A A A A A A A A A A A A A A . A A . .    
410	. C C C C C C C C C C C C C C C C C C C . C C . .    
411	. T T T T T T T T T T T T T T T T T T T . T T . .    
412	. C C C C C C C C C C C C C C C C C C C . C C . .    
413	. A A A A A A A A A A A A A A A A A A A . A A . .    
414	. T T T T T T T T T T T T T T T T T T T . T T . .    
415	. A A A A A A A A A A A A A A A A A A A . A A . .    
416	. C C C C C C C C C C C C C C C C C C C . C C . .    
417	. A A A A A A A A A A A A A A A A A A A . A A . .    
418	. T T T T T T T T T T T T T T T T T T T . T T . .    
419	. T T T T T T T T T T T T T T T T T T T . T T . .    
420	. - - - T - - - - - - - - - - - - - - - . - - . .    
421	. G G G G G G G G G G G G G G G G G G G . G G . .    
422	. A A A A A A A A A A A A A A A A A A A . A A . .    
423	. T T T T T T T T T T T T T T T T T T T . T T . .    
424	. T T T T T T T T T T T T T T T T T T T . T T . .    
425	. T T T T T T T T T T T T T T T T T T T . T T . .    
426	. G G G G G G G G G G G G G G G G G G G . G G . .    
427	. T T T T T T T T T T T T T T T T T T T . T T . .    
428	. T T T T T T T T T T T T T T T T T T T . T T . .    
429	. G G G G G G G G G G G G G G G G G G G . G G . .    
430	. A A A A A A A A A A A A A A A A A A A . A A . .    
431	. G G G G G G G G G G G G G G G G G G G . G G . .    
432	. C C C C C C C C C C C C C C C C C C C . C C . .    
433	. A A A A A A A A A A A A A A A A A A A . A A . .    
434	. G G G G G G G G G G G G G G G G G G G . G G . .    
435	. A A A A A A A A A A A A A A A A A A A . A A . .    
436	. G G G G G G G G G G G G G G G G G G G . G G . .    
437	. C C C C C C C C C C C C C C C C C C C . C C . .    
438	. T T T T T T T T T T T T T T T T T T T . T T . .    
439	. A A A A A A A A A A A A A A A A A A A . A A . .    
440	. A A A A A A A A A A A A A A A A A A A . A A . .    
441	. C C C C C C C C C C C C C C C C C C C . C C . .    
442	. A A A A A A A A A A A A A A A A A A A . A A . .    
443	. A A A A A A A A A A A A A A A A A A A . A A . .    
444	. T T T T T T T T T T T T T T T T T T T . T T . .    
445	. A A A A A A A A A A A A A A A A A A A . A A . .    
446	. A A A A A A A A A A A A A A A A A A A . A A . .    
447	. G G G G G G G G G G G G G G G G G G G . G G . .    
448	. C C C C C C C C C C C C C C C C C C C . C C . .    
449	. A A A A A A A A A A A A A A A A A A A . A A . .    
450	. T T T T T T T T T T T T T T T T T T T T T T . .    
451	. G G G G G G G G G G G G G G G G G G G G G G . .    
452	. T T T T T T T T T T C T T T T T T T T T T T . .    
453	. T T T T T T T T T T T T T T T T T T T T T T . .    
454	. T T T T T T T T T T T T T T T T T T T T T T . .    
455	. A A A A A A A A A A A A A A A A A A A A A A . .    
456	. A A A A A A A A A A A A A A A A A A A A A A . .    
457	. C C C C C C C C C C C C C C C C C C C C C C . .    
458	. A A A A A A A A A A A A A A A A A A A A A A . .    
459	. A A A A A A A A A A A A A A A A A A A A A A . .    
460	. G G G G G G G G G G G G G G G G G G G G G G . .    
461	. C C C C C C C C C C C C C C C C C C C C C C . .    
462	. A A A A A A A A A A A A A A A A A A A A A A . .    
463	. C C C C C C C C C C C C C C C C C C C C C C . .    
464	. T T T T T T T T T T T T T T T T T T T T T T . .    
465	. G G G G G G G G G G G G G G G G G G G G G G . .    
466	. A A A A A A A A A A A A A A A A A A A A A A . .    
467	. G G G G G G G G G G G G G G G G G G G G G G . .    
468	. G T T T T T T T T T T T T T T T T T T T T T . .    
469	. . G G G G G G G G G G G G G G G G G G G G G . .    
470	. . A A A A A A A A A A A A A A A A A A A A A . .    
471	. . T T T T T T T T T T T T T T T T T T T T T . .    
472	. . T T T T T T T T T T T T T T T T T T T T T . .    
473	. . G G G G G G G G G G G G G G G G G G G G G . .    
474	. . A A A A A A A A A A A A A A A A A A A A A . .    
475	. . G G G G G G G G G G G G G G G G G G G G G . .    
476	. . G G G G G G G G G G G G G G G G G G G G G . .    
477	. . A A A A A A A A A A A A A A A A A A A A A . .    
478	. . T T T T T T T T T T T T T T T T T T T T T . .    
479	. . A A A A A A A A A A A A A A A A A A A A A . .    
480	. . A A A A A A A A A A A A A A A A A A A A A . .    
481	. . G G G G G G G G G G G G G G G G G G G G G . .    
482	. . A A A A A A A A A A A A A A A A A A A A A . .    
483	. . G G G G G G G G G G G G G G G G G G G G G . .    
484	. . C C C C C C C C C C C C C C C C C C C C C . .    
485	. . A A A A A A A A A A A A A A A A A A A A A . .    
486	. . T T T T T T T T T T T T T T T T T T T T T . .    
487	. . A A A A A A A A A A A A A A A A A A A A A . .    
488	. . T T T T T T T T T T T T T T T T T T T T T . .    
489	. . T T T T T T T T T T T T T T T T T T T T T . .    
490	. . A A A A A A A A A A A A A A A A A A A A A . .    
491	. . G G G G G G G G G G G G G G G G G G G G G . .    
492	. . A A A A A A A A A A A A A A A A A A A A A . .    
493	. . A A A A A A A A A A A A A A A A A A A A A . .    
494	. . A A A A A A A A A A A A A A A A A A A A A . .    
495	. . A A A A A A A A A A A A A A A A A A A A A . .    
496	. . C C C C C C C C C C C C C C C C C C C C C . .    
497	. . A A A A A A A A A A A A A A A A A A A A A . .    
498	. . T T T T T T T T T T T T T T T T T T T T T . .    
499	. . G G G G G G G G G G G G G G G G G G G G G . .    
500	. . G G G G G G G G G G G G G G G G G G G G G . .    
501	. . T T T T T T T T T T T T T T T T T T T T T . .    
502	. . C C C C C C C C C C C C C C C C C C C C C . .    
503	. . A A A A A A A A A A A A A A A A A A A A A . .    
504	. . A A A A A A A A A A A A A A A A A A A A A . .    
505	. . A A A A A A A A A A A A A A A A A A A A A . .    
506	. . C C C C C C C C C C C C C C C C C C C C C . .    
507	. . A A A A A A A A A A A A A A A A A A A A A . .    
508	. . A A A A A A A A A A A A A A A A A A A A A . .    
509	. . G G G G G G G G G G G G G G G G G G G G G . .    
510	. . T T T T T T T T T T T T T T T T T T T T T . .    
511	. . C C C C C C C C C C C C C C C C C C C C C . .    
512	. . A A A A A A A A A A A A A A A A A A A A A . .    
513	. . T T T T T T T T T T T T T T T T T T T T T . .    
514	. . A A A A A A A A A A A A A A A A A A A A A . .    
515	. . A A A A A A A A A A A A A A A A A A A A A . .    
516	. . A A A A A A A A A A A A A A A A A A A A A . .    
517	. . G G G G G G G G G G G G G G G G G G G G G . .    
518	. . C C C C C C C C C C C C C C C C C C C C C . .    
519	. . G G G G G G G G G G G G G G G G G G G G G . .    
520	. . T T T T T T T T T T T T T T T T T T T T T . .    
521	. . T T T T T T T T T T T T T T T T T T T T T . .    
522	. . T T T T T T T T T T T T T T T T T T T T T . .    
523	. . C C C C C C C C C C C C C C C C C C C C C . .    
524	. . A A A A A A A A A A A A A A A A A A A A A . .    
525	. . G G G G G G G G G G G G G G G G G G G G G . .    
526	. . G G G G G G G G G G G G G G G G G G G G G . .    
527	. . T T T T T T T T T T T T T T T T T T T T T . .    
528	. . T T T T T T T T T T T T T T T T T T T T T . .    
529	. . A A A A A A A A A A A A A A A A A A A A A . .    
530	. . C C C C C C C C C C C C C C C C C C C C C . .    
531	. . A A A A A A A A A A A A A A A A A A A A A . .    
532	. . T T T T T T T T T T T T T T T T T T T T T . .    
533	. . T T T T T T T T T T T T T T T T T T T T T . .    
534	. . A A A A A A A A A A A A A A A A A A A A A . .    
535	. . A A A A A A A A A A A A A A A A A A A A A . .    
536	. . T T T T T T T T T T T T T T T T T T T T T . .    
537	. . G G G G G G G G G G G G G G G G G G G G G . .    
538	. . G G G G G G G G G G G G G G G G G G G G G . .    
539	. . A A A A A A A A A A A A A A A A A A A A A . .    
540	. . T T T T T T T T T T T T T T T T T T T T T . .    
541	. . G G G G G G G G G G G G G G G G G G G G G . .    
542	. . C C C C C C C C C C C C C C C C C C C C C . .    
543	. . A A A A A A A A A A A A A A A A A A A A A . .    
544	. . T T T T T T T T T T T T T T T T T T T T T . .    
545	. . G G G G G G G G G G G G G G G G G G G G G . .    
546	. . A A A A A A A A A A A A A A A A A A A A A . .    
547	. . A A A A A A A A A A A A A A A A A A A A A . .    
548	. . T T T T T T T T T T T T T T T T T T T T T . .    
549	. . C C C C C C C C C C C C C C C C C C C C C . .    
550	. . A A A A A A A A A A A A A A A A A A A A A . .    
551	. . G G G G G G G G G G G G G G G G G G G G G . .    
552	. . T T T T T T T T T T T T T T T T T T T T T . .    
553	. . G G G G G G G G G G G G G G G G G G G G G . .    
554	. . A A A A A A A A A A A A A A A A A A A A A . .    
555	. . C C C C C C C C C C C C C C C C C C C C C . .    
556	. . G G G G G G G G G G G G G G G G G G G G G . .    
557	. . G G G G G G G G G G G G G G G G G G G G G . .    
558	. . T T T T T T T T T T T T T T T T T T T T T . .    
559	. . T T T T T T T T T T T T T T T T T T T T T . .    
560	. . C C . C C C C C C C C C C C C C C C C C C . .    
561	. . A A . A A A A A A A A A A A A A A A A A A . .    
562	. . C C . C C C C C C C C C C C C C C C C C C . .    
563	. . T T . T T T T T T T T T T T T T T T T T T . .    
564	. . T T . T T T T T T T T T T T T T T T T T T . .    
565	. . T T . T T T T T T T T T T T T T T T T T T . .    
566	. . C C . C C C C C C C C C C C C C C C C C C . .    
567	. . A A . A A A A A A A A A A A A A A A A A A . .    
568	. . A A . A A A A A A A A A A A A A A A A A A . .    
569	. . G G . G G G G G G G G G G G G G G G G G G . .    
570	. . T T . T T T T T T T T T T T T T T T T T T . .    
571	. . A A . A A A A A A A A A A A A A A A A A A . .    
572	. . T T . T T T T T T T T T T T T T T T T T T . .    
573	. . T T . T T T T T T T T T T T T T T T T T T . .    
574	. . T T . T T T T T T T T T T T T T T T T T T . .    
575	. . A A . A A A A A A A A A A A A A A A A A A A .    
576	. . . . . A A A A A A A A A A A A A A A A A A A .    
577	. . . . . G G G G G G G G G G G G G G G G G G G .    
578	. . . . . A A A A A A A A A A A A A A A A A A A .    
579	. . . . . T T T T T T T T T T T T T T T T T T T .    
580	. . . . . C C C C C C C C C C C C C C C C C C C .    
581	. . . . . A A A A A A A A A A A A A A A A A A A .    
582	. . . . . C C C C C C C C C C C C C C C C C C C .    
583	. . . . . A A A A A A A A A A A A A A A A A A A .    
584	. . . . . G G G G G G G G G G G G G G G G G G G .    
585	. . . . . C C C C C C C C C C C C C C C C C C C .    
586	. . . . . T T T T T T T T T T T T T T T T T T T .    
587	. . . . . T T T T T T T T T T T T T T T T T T T .    
588	. . . . . C C C C C C C C C C C C C C C C C C C .    
589	. . . . . G G G G G G G G G G G G G G G G G G G .    
590	. . . . . G G G G G G G G G G G G G G G G G G G .    
591	. . . . . T T T T T T T T T T T T T T T T T T T .    
592	. . . . . G G G G G G G G G G G G G G G G G G G .    
593	. . . . . A A A A A A A A A A A A A A A A A A A .    
594	. . . . . T T T T T T T T T T T T T T T T T T T .    
595	. . . . . T T T T T T T T T T T T T T T T T T T .    
596	. . . . . A A A A A A A A A A A A A A A A A A A .    
597	. . . . . C C C C C C C C C C C C C C C C C C C .    
598	. . . . . A A A A A A A A A A A A A A A A A A A .    
599	. . . . . T T T T T T T T T T T T T T T T T T T .    
600	. . . . . G G G G G G G G G G G G G G G G G G G .    
601	. . . . . G G G G G G G G G G G G G G G G G G G .    
602	. . . . . A A A A A A A A A A A A A A A A A A A .    
603	. . . . . C C C C C C C C C C C C C C C C C C C .    
604	. . . . . T T T T T T T T T T T T T T T T T T T .    
605	. . . . . T T T T T T T T T T T T T T T T T T T .    
606	. . . . . T T T T T T T T T T T T T T T T T T T .    
607	. . . . . T T T T T T T T T T T T T T T T T T T .    
608	. . . . . A A A A A A A A A A A A A A A A A A A .    
609	. . . . . T T T T T T T T T T T T T T T T T T T .    
610	. . . . . T T T T T T T T T T T T T T T T T T T .    
611	. . . . . G G G G G G G G G G G G G G G G G G G .    
612	. . . . . T T T T T T T T T T T T T T T T T T T .    
613	. . . . . G G G G G G G G G G G G G G G G G G G .    
614	. . . . . A A A A A A A A A A A A A A A A A A A .    
615	. . . . . G G G G G G G G G G G G G G G G G G G .    
616	. . . . . T T T T T T T T T T T T T T T T T T T .    
617	. . . . . C C C C C C C C C C C C C C C C C C C .    
618	. . . . . A A A A A A A A A A A A A A A A A A A .    
619	. . . . . T T T T T T T T T T T T T T T T T T T .    
620	. . . . . T T T T T T T T T T T T T T T T T T T .    
621	. . . . . T T T T T T T T T T T T T T T T T T T .    
622	. . . . . T T T T T T T T T T T T T T T T T T T .    
623	. . . . . A A A A A A A A A A A A A A A A A A A .    
624	. . . . . T T T T T T T T T T T T T T T T T T T .    
625	. . . . . T T T T T T T T T T T T T T T T T T T .    
626	. . . . . A A A A A A A A A A A A A A A A A A A .    
627	. . . . . G G G G G G G G G G G G G G G G G G G .    
628	. . . . . G G G G G G G G G G G G G G G G G G G .    
629	. . . . . T T T T T T T T T T T T T T T T T T T .    
630	. . . . . C C C C C C C C C C C C C C C C C C C .    
631	. . . . . A A A A A A A A A A A A A A A A A A A .    
632	. . . . . A A A A A A A A A A A A A A A A A A A .    
633	. . . . . A A A A A A A A A A A A A A A A A A A .    
634	. . . . . G G G G G G G G G G G G G G G G G G G .    
635	. . . . . T T T T T T T T T T T T T T T T T T T .    
636	. . . . . T T T T T T T T T T T T T T T T T T T .    
637	. . . . . G G G G G G G G G G G G G G G G G G G .    
638	. . . . . A A A A A A A A A A A A A A A A A A A .    
639	. . . . . T T T T T T T T T T T T T T T T T T T .    
640	. . . . . T T T T T T T T T T T T T T T T T T T .    
641	. . . . . C C C C C C C C C C C C C C C C C C C .    
642	. . . . . T T T T T T T T T T T T T T T T T T T .    
643	. . . . . C C C C C C C C C C C C C C C C C C C .    
644	. . . . . A A A A A A A A A A A A A A A A A A A .    
645	. . . . . C C C C C C C C C C C C C C C C C C C .    
646	. . . . . A A A A A A A A A A A A A A A A A A A .    
647	. . . . . T T T T T T T T T T T T T T T T T T T .    
648	. . . . . T T T T T T T T T T T T T T T T T T T .    
649	. . . . . A A A A A A A A A A A A A A A A A A A .    
650	. . . . . A A A A A A A A A A A A A A A A A A A .    
651	. . . . . A A A A A A A A A A A A A A A A A A A .    
652	. . . . . T T T T T T T T T T T T T T T T T T T .    
653	. . . . . G G G G G G G G G G G G G G G G G G G .    
654	. . . . . A A A A A A A A A A A A A A A A A A A .    
655	. . . . . A A A A A A A A A A A A A A A A A A A .    
656	. . . . . A A A A A A A A A A A A A A A A A A A .    
657	. . . . . C C C C C C C C C C C C C C C C C C C .    
658	. . . . . A A A A A A A A A A A A A A A A A A A .    
659	. . . . . T T T T T T T T T T T T T T T T T T T .    
660	. . . . . G G G G G G G G G G G G G G G G G G G .    
661	. . . . . G G G G G G G G G G G G G G G G G G G .    
662	. . . . . T T T T T T T T T T T T T T T T T T T .    
663	. . . . . C C C C C C C C C C C C C C C C C C C .    
664	. . . . . C C C C C C C C C C C C C C C C C C C .    
665	. . . . . A A A A A A A A A A A A A A A A A A A .    
666	. . . . . . T T T T T T T T T T T T T T T T T T .    
667	. . . . . . C C C C C C C C C C C C C C C C C C .    
668	. . . . . . G G G G G G G G G G G G G G G G G G .    
669	. . . . . . G G G G G G G G G G G G G G G G G G .    
670	. . . . . . C C C C C C C C C C C C C C C C C C .    
671	. . . . . . T T T T T T T T T T T T T T T T T T .    
672	. . . . . . T T T T T T T T T T T T T T T T T T .    
673	. . . . . . A A A A A A A A A A A A A A A A A A A    
674	. . . . . . A A A A A A A A A A A A A A A A A A A    
675	. . . . . . A A A A A A A A A A A A A A A A A A A    
676	. . . . . . A A A A A A A A A A A A A A A A A A A    
677	. . . . . . A A A A A A A A A A A A A A A A A A A    
678	. . . . . . T T T T T T T T T T T T T T T T T T T    
679	. . . . . . G G G G G G G G G G G G G G G G G G G    
680	. . . . . . A A A A A A A A A A A A A A A A A A A    
681	. . . . . . A A A A A A A A A A A A A A A A A A A    
682	. . . . . . T T T T T T T T T T T T T T T T T T T    
683	. . . . . . G G G G G G G G G G G G G G G G G G G    
684	. . . . . . C C C C C C C C C C C C C C C C C C C    
685	. . . . . . T . T T T T T T T T T T T T T T T T T    
686	. . . . . . T . T T T T T T T T T T T T T T T T T    
687	. . . . . . G . G G G G G G G G G G G G G G G G G    
688	. . . . . . T . T T T T T T T T T T T T T T T T T    
689	. . . . . . T . T T T T T T T T T T T T T T T T T    
690	. . . . . . G . G G G G G G G G G G G G G G G G G    
691	. . . . . . T . T T T T T T T T T T T T T T T T T    
692	. . . . . . G . G G G G G G G G G G G G G G G G G    
693	. . . . . . A . A A A A A A A A A A A A A A A A A    
694	. . . . . . T . T T T T T T T T T T T T T T T T T    
695	. . . . . . T . T T T T T T T T T T T T T T T T T    
696	. . . . . . A . A A A A A A A A A A A A A A A A A    
697	. . . . . . T . T T T T T T T T T T T T T T T T T    
698	. . . . . . C . C C C C C C C C C C C C C C C C C    
699	. . . . . . T . T T T T T T T T T T T T T T T T T    
700	. . . . . . G . G G G G G G G G G G G G G G G G G    
701	. . . . . . G . G G G G G G G G G G G G G G G G G    
702	. . . . . . T . T T T T T T T T T T T T T T T T T    
703	. . . . . . A . A A A A A A A A A A A A A A A A A    
704	. . . . . . A . A A A A A A A A A A A A A A A A A    
705	. . . . . . C . C C C C C C C C C C C C C C C C C    
706	. . . . . . C . C C C C C C C C C C C C C C C C C    
707	. . . . . . T . T T T T T T T T T T T T T T T T T    
708	. . . . . . G . G G G G G G G G G G G G G G G G G    
709	. . . . . . C . C C C C C C C C C C C C C C C C C    
710	. . . . . . T . T T T T T T T T T T T T T T T T T    
711	. . . . . . T . T T T T T T T T T T T T T T T T T    
712	. . . . . . C . C C C C C C C C C C C C C C C C C    
713	. . . . . . T . T T T T T T T T T T T T T T T T T    
714	. . . . . . A . A A A A A A A A A A A A A A A A A    
715	. . . . . . T . T T T T T T T T T T T T T T T T T    
716	. . . . . . T . T T T T T T T T T T T T T T T T T    
717	. . . . . . A . A A A A A A A A A A A A A A A A A    
718	. . . . . . T . T T T T T T T T T T T T T T T T T    
719	. . . . . . . . T T T T T T T T T T T T T T T T T    
720	. . . . . . . . T T T T T T T T T T T T T T T T T    
721	. . . . . . . . A A A A A A A A A A A A A A A A A    
722	. . . . . . . . G G G G G G G G G G G G G G G G G    
723	. . . . . . . . G G G G G G G G G G G G G G G G G    
724	. . . . . . . . A A A A A A A A A A A A A A A A A    
725	. . . . . . . . A A A A A A A A A A A A A A A A A    
726	. . . . . . . . A A A A A A A A A A A A A A A A A    
727	. . . . . . . . G A A A A A A A A A A A A A A A A    
728	. . . . . . . . . G G G G G G G G G G G G G G G G    
729	. . . . . . . . . G G G G G G G G G G G G G G G G    
730	. . . . . . . . . C C C C C C C C C C C C C C C C    
731	. . . . . . . . . T T T T T T T T T T T T T T T T    
732	. . . . . . . . . T T T T T T T T T T T T T T T T    
733	. . . . . . . . . C C C C C C C C C C C C C C C C    
734	. . . . . . . . . A A A A A A A A A A A A A A A A    
735	. . . . . . . . . A A A A A A A A A A A A A A A A    
736	. . . . . . . . . A A A A A A A A A A A A A A A A    
737	. . . . . . . . . . A A A A A A A A A A A A A A A    
738	. . . . . . . . . . G G . G G G G G G G G G G G G    
739	. . . . . . . . . . T T . T T T T T T T T T T T T    
740	. . . . . . . . . . A A . A A A A A A A A A A A A    
741	. . . . . . . . . . C C . C C C C C C C C C C C C    
742	. . . . . . . . . . A C . A A A A A A A A A A A A    
743	. . . . . . . . . . A . . A A A A A A A A A A A A    
744	. . . . . . . . . . A . . A A A A A A A A A A A A    
745	. . . . . . . . . . A . . A A A A A A A A A A A A    
746	. . . . . . . . . . C . . C C C C C C C C C C C C    
747	. . . . . . . . . . A . . A A A A A A A A A A A A    
748	. . . . . . . . . . T . . T T T T T T T T T T T T    
749	. . . . . . . . . . T . . T T T T T T T T T T T T    
750	. . . . . . . . . . G . . G G G G G G G G G G G G    
751	. . . . . . . . . . C . . C C C C C C C C C C C C    
752	. . . . . . . . . . A . . A A A A A A A A A A A A    
753	. . . . . . . . . . A . . A A A A A A A A A A A A    
754	. . . . . . . . . . C . . C C C C C C C C C C C C    
755	. . . . . . . . . . T . . T T T T T T T T T T T T    
756	. . . . . . . . . . G . . G G G G G G G G G G G G    
757	. . . . . . . . . . A . . A A A A A A A A A A A A    
758	. . . . . . . . . . T . . A A A A A A A A A A A A    
759	. . . . . . . . . . . . . T T T T T T T T T T T T    
760	. . . . . . . . . . . . . T T T T T T T T T T T T    
761	. . . . . . . . . . . . . T T T T T T T T T T T T    
762	. . . . . . . . . . . . . T T T T T T T T T T T T    
763	. . . . . . . . . . . . . G G G G G G G G G G G G    
764	. . . . . . . . . . . . . T T T T T T T T T T T T    
765	. . . . . . . . . . . . . A A A A A A A A A A A A    
766	. . . . . . . . . . . . . G G G G G G G G G G G G    
767	. . . . . . . . . . . . . A A A A A A A A A A A A    
768	. . . . . . . . . . . . . A A A A A A A A A A A A    
769	. . . . . . . . . . . . . A A A A A A A A A A A A    
770	. . . . . . . . . . . . . A A A A A A A A A A A A    
771	. . . . . . . . . . . . . A A G A A A A A A A A A    
772	. . . . . . . . . . . . . C C C C C C C C C C C C    
773	. . . . . . . . . . . . . A A A A A A A A A A A A    
774	. . . . . . . . . . . . . T T T T T T T T T T T T    
775	. . . . . . . . . . . . . A A A A A A A A A A A A    
776	. . . . . . . . . . . . . - T T - T - - T - - T T  6	 indel  
777	. . . . . . . . . . . . . - A A - A - - A - - A A  6	 indel  
778	. . . . . . . . . . . . . T T T T T T T T T T T T    
779	. . . . . . . . . . . . . T T T T T T T T T T T T    
780	. . . . . . . . . . . . . G G G G G G G G G G G G    
781	. . . . . . . . . . . . . C C C C C C C C C C C C    
782	. . . . . . . . . . . . . T T T T T T T T T T T T    
783	. . . . . . . . . . . . . T T T T T T T T T T T T    
784	. . . . . . . . . . . . . G G G G G G G G G G G G    
785	. . . . . . . . . . . . . T T T T T T T T T T T T    
786	. . . . . . . . . . . . . T T T T T T T T T T T T    
787	. . . . . . . . . . . . . T T T T T T T T T T T T    
788	. . . . . . . . . . . . . T T T T T T T T T T T T    
789	. . . . . . . . . . . . . G G G G G G G G G G G G    
790	. . . . . . . . . . . . . A . A A A A A A A A A A    
791	. . . . . . . . . . . . . G . G G G G G G G G G G    
792	. . . . . . . . . . . . . A . A A A A A A A A A A    
793	. . . . . . . . . . . . . A . A A A A A A A A A A    
794	. . . . . . . . . . . . . T . T T T T T T T T T T    
795	. . . . . . . . . . . . . G . G G G G G G G G G G    
796	. . . . . . . . . . . . . T . T T T T T T T T T T    
797	. . . . . . . . . . . . . G . G G G G G G G G G G    
798	. . . . . . . . . . . . . A . A A A A A A A A A A    
799	. . . . . . . . . . . . . T . T T T T T T T T T T    
800	. . . . . . . . . . . . . G . G G G G G G G G G G    
801	. . . . . . . . . . . . . T . T T T T T T T T T T    
802	. . . . . . . . . . . . . . . A A A A A A A A A A    
803	. . . . . . . . . . . . . . . G G G G G G G G G G    
804	. . . . . . . . . . . . . . . A A A A A A A A A A    
805	. . . . . . . . . . . . . . . T T T T T T T T T T    
806	. . . . . . . . . . . . . . . G G G G G G G G G G    
807	. . . . . . . . . . . . . . . T T T T T T T T T T    
808	. . . . . . . . . . . . . . . A A A A A A A A A A    
809	. . . . . . . . . . . . . . . G G G G G G G G G G    
810	. . . . . . . . . . . . . . . A A A A A A A A A A    
811	. . . . . . . . . . . . . . . A A A A A A A A A A    
812	. . . . . . . . . . . . . . . A A A A A A A A A A    
813	. . . . . . . . . . . . . . . A A A A A A A A A A    
814	. . . . . . . . . . . . . . . T T T T T T T T T T    
815	. . . . . . . . . . . . . . . G G G G G G G G G G    
816	. . . . . . . . . . . . . . . C C C C C C C C C C    
817	. . . . . . . . . . . . . . . C C C C C C C C C C    
818	. . . . . . . . . . . . . . . A A A A A A A A A A    
819	. . . . . . . . . . . . . . . C C C C C C C C C C    
820	. . . . . . . . . . . . . . . G G G G G G G G G G    
821	. . . . . . . . . . . . . . . G G G G G G G G G G    
822	. . . . . . . . . . . . . . . A A A A A A A A A A    
823	. . . . . . . . . . . . . . . T T T T T T T T T T    
824	. . . . . . . . . . . . . . . T T T T T T T T T T    
825	. . . . . . . . . . . . . . . G G G G G G G G G G    
826	. . . . . . . . . . . . . . . C C C C C C C C C C    
827	. . . . . . . . . . . . . . . A A A A A A A A A A    
828	. . . . . . . . . . . . . . . A A A A A A A A A A    
829	. . . . . . . . . . . . . . . T T T T T T T T T T    
830	. . . . . . . . . . . . . . . A A A A A A A A A A    
831	. . . . . . . . . . . . . . . C C C C C C C C C C    
832	. . . . . . . . . . . . . . . G G G G G G G G G G    
833	. . . . . . . . . . . . . . . T T T T T T T T T T    
834	. . . . . . . . . . . . . . . C C C C C C C C C C    
835	. . . . . . . . . . . . . . . C A A A A A A A A A    
836	. . . . . . . . . . . . . . . . T T T T T T T T T    
837	. . . . . . . . . . . . . . . . G G G G G G G G G    
838	. . . . . . . . . . . . . . . . A A A A A A A A A    
839	. . . . . . . . . . . . . . . . T T T T T T T T T    
840	. . . . . . . . . . . . . . . . T T T T T T T T T    
841	. . . . . . . . . . . . . . . . C C C C C C C C C    
842	. . . . . . . . . . . . . . . . C C C C C C C C C    
843	. . . . . . . . . . . . . . . . A A A A A A A A A    
844	. . . . . . . . . . . . . . . . A A A A A A A A A    
845	. . . . . . . . . . . . . . . . T T T T T T T T T    
846	. . . . . . . . . . . . . . . . A A A A A A A A A    
847	. . . . . . . . . . . . . . . . T T T T T T T T T    
848	. . . . . . . . . . . . . . . . G G G G G G G G G    
849	. . . . . . . . . . . . . . . . C C C C C C C C C    
850	. . . . . . . . . . . . . . . . A A A A A A A A A    
851	. . . . . . . . . . . . . . . . A A A A A A A A A    
852	. . . . . . . . . . . . . . . . C C C C C C C C C    
853	. . . . . . . . . . . . . . . . G G G G G G G G G    
854	. . . . . . . . . . . . . . . . T T T T T T T T T    
855	. . . . . . . . . . . . . . . . T T T T T T T T T    
856	. . . . . . . . . . . . . . . . A A A A A A A A A    
857	. . . . . . . . . . . . . . . . C C C C C C C C C    
858	. . . . . . . . . . . . . . . . T T T T T T T T T    
859	. . . . . . . . . . . . . . . . T T T T T T T T T    
860	. . . . . . . . . . . . . . . . C C C C C C C C C    
861	. . . . . . . . . . . . . . . . A A A A A A A A A    
862	. . . . . . . . . . . . . . . . A A A A A A A A A    
863	. . . . . . . . . . . . . . . . A A A A A A A A A    
864	. . . . . . . . . . . . . . . . T T T T T T T T T    
865	. . . . . . . . . . . . . . . . G G G G G G G G G    
866	. . . . . . . . . . . . . . . . C C C C C C C C C    
867	. . . . . . . . . . . . . . . . A A A A A A A A A    
868	. . . . . . . . . . . . . . . . G G G G G G G G G    
869	. . . . . . . . . . . . . . . . G G G G G G G G G    
870	. . . . . . . . . . . . . . . . C C C C C C C C C    
871	. . . . . . . . . . . . . . . . G G G G G G G G G    
872	. . . . . . . . . . . . . . . . C C C C C C C C C    
873	. . . . . . . . . . . . . . . . C C C C C C C C C    
874	. . . . . . . . . . . . . . . . C C C C C C C C C    
875	. . . . . . . . . . . . . . . . A A A A A A A A A    
876	. . . . . . . . . . . . . . . . T T T T T T T T T    
877	. . . . . . . . . . . . . . . . T T T T T T T T T    
878	. . . . . . . . . . . . . . . . G G G G G G G G G    
879	. . . . . . . . . . . . . . . . C C C C C C C C C    
880	. . . . . . . . . . . . . . . . T T T T T T T T T    
881	. . . . . . . . . . . . . . . . T T T T T T T T T    
882	. . . . . . . . . . . . . . . . A A A A A A A A A    
883	. . . . . . . . . . . . . . . . T T T T T T T T T    
884	. . . . . . . . . . . . . . . . A A A A A A A A A    
885	. . . . . . . . . . . . . . . . G G G G G G G G G    
886	. . . . . . . . . . . . . . . . G G G G G G G G G    
887	. . . . . . . . . . . . . . . . A A A A A A A A A    
888	. . . . . . . . . . . . . . . . T T T T T T T T T    
889	. . . . . . . . . . . . . . . . G G G G G G G G G    
890	. . . . . . . . . . . . . . . . G G G G G G G G G    
891	. . . . . . . . . . . . . . . . G G G G G G G G G    
892	. . . . . . . . . . . . . . . . A A A A A A A A A    
893	. . . . . . . . . . . . . . . . C C C C C C C C C    
894	. . . . . . . . . . . . . . . . G G G G G G G G G    
895	. . . . . . . . . . . . . . . . G G G G G G G G G    
896	. . . . . . . . . . . . . . . . T T T T T T T T T    
897	. . . . . . . . . . . . . . . . C C C C C C C C C    
898	. . . . . . . . . . . . . . . . A A A A A A A A A    
899	. . . . . . . . . . . . . . . . A A A A A A A A A    
900	. . . . . . . . . . . . . . . . C C C C C C C C C    
901	. . . . . . . . . . . . . . . . A A A A A A A A A    
902	. . . . . . . . . . . . . . . . A A A A A A A A A    
903	. . . . . . . . . . . . . . . . G G G G G G G G G    
904	. . . . . . . . . . . . . . . . T T T T T T T T T    
905	. . . . . . . . . . . . . . . . C C C C C C C C C    
906	. . . . . . . . . . . . . . . . G G G G G G G G G    
907	. . . . . . . . . . . . . . . . G G G G G G G G G    
908	. . . . . . . . . . . . . . . . T T T T T T T T T    
909	. . . . . . . . . . . . . . . . G G G G G G G G G    
910	. . . . . . . . . . . . . . . . G G G G G G G G G    
911	. . . . . . . . . . . . . . . . C C C C C C C C C    
912	. . . . . . . . . . . . . . . . G G G G G G G G G    
913	. . . . . . . . . . . . . . . . A A A A A A A A A    
914	. . . . . . . . . . . . . . . . C C C C C C C C C    
915	. . . . . . . . . . . . . . . . G G G G G G G G G    
916	. . . . . . . . . . . . . . . . T T T T T T T T T    
917	. . . . . . . . . . . . . . . . C T C C C C C C C    
918	. . . . . . . . . . . . . . . . A A A A A A A A A    
919	. . . . . . . . . . . . . . . . C C C C C C C C C    
920	. . . . . . . . . . . . . . . . A A A A A A A A A    
921	. . . . . . . . . . . . . . . . T T T T T T T T T    
922	. . . . . . . . . . . . . . . . T T T T T T T T T    
923	. . . . . . . . . . . . . . . . C C C C C C C C C    
924	. . . . . . . . . . . . . . . . T T T T T T T T T    
925	. . . . . . . . . . . . . . . . G G G G G G G G G    
926	. . . . . . . . . . . . . . . . C C C C C C C C C    
927	. . . . . . . . . . . . . . . . T T T T T T T T T    
928	. . . . . . . . . . . . . . . . G G G G G G G G G    
929	. . . . . . . . . . . . . . . . A A A A A A A A A    
930	. . . . . . . . . . . . . . . . C T C C T C C C T    
931	. . . . . . . . . . . . . . . . A A A A A A A A A    
932	. . . . . . . . . . . . . . . . G G G G G G G G G    
933	. . . . . . . . . . . . . . . . T T T T T T T T T    
934	. . . . . . . . . . . . . . . . G G G G G G G G G    
935	. . . . . . . . . . . . . . . . G G G G G G G G G    
936	. . . . . . . . . . . . . . . . G G G G G G G G G    
937	. . . . . . . . . . . . . . . . A A A A A A A A A    
938	. . . . . . . . . . . . . . . . C C C C C C C C C    
939	. . . . . . . . . . . . . . . . G G G G G G G G G    
940	. . . . . . . . . . . . . . . . C C C C C C C C C    
941	. . . . . . . . . . . . . . . . A A A A A A A A A    
942	. . . . . . . . . . . . . . . . G G G G G G G G G    
943	. . . . . . . . . . . . . . . . T T T T T T T T T    
944	. . . . . . . . . . . . . . . . C C C C C C C C C    
945	. . . . . . . . . . . . . . . . T T T T T T T T T    
946	. . . . . . . . . . . . . . . . C C C C C C C C C    
947	. . . . . . . . . . . . . . . . T T T T T T T T T    
948	. . . . . . . . . . . . . . . . T T T T T T T T T    
949	. . . . . . . . . . . . . . . . C C C C C C C C C    
950	. . . . . . . . . . . . . . . . C C C C C C C C C    
951	. . . . . . . . . . . . . . . . C T T T T T T T T    
952	. . . . . . . . . . . . . . . . . C C C C C C C C    
953	. . . . . . . . . . . . . . . . . G G G G G G G G    
954	. . . . . . . . . . . . . . . . . A A A A A A A A    
955	. . . . . . . . . . . . . . . . . A A A A A A A A    
956	. . . . . . . . . . . . . . . . . G G G G G G G G    
957	. . . . . . . . . . . . . . . . . A A A A A A A A    
958	. . . . . . . . . . . . . . . . . A A A A A A A A    
959	. . . . . . . . . . . . . . . . . C C C C C C C C    
960	. . . . . . . . . . . . . . . . . C C C C C C C C    
961	. . . . . . . . . . . . . . . . . A A A A A A A A    
962	. . . . . . . . . . . . . . . . . A A A A A A A A    
963	. . . . . . . . . . . . . . . . . C C C C C C C C    
964	. . . . . . . . . . . . . . . . . T T T T T T T T    
965	. . . . . . . . . . . . . . . . . T T T T T T T T    
966	. . . . . . . . . . . . . . . . . A A A A A A A A    
967	. . . . . . . . . . . . . . . . . G G G G G G G G    
968	. . . . . . . . . . . . . . . . . G G G G G G G G    
969	. . . . . . . . . . . . . . . . . G C G G G G G G    
970	. . . . . . . . . . . . . . . . . G . G G G G G G    
971	. . . . . . . . . . . . . . . . . A . A A A A A A    
972	. . . . . . . . . . . . . . . . . A . A A A A A A    
973	. . . . . . . . . . . . . . . . . G . G G G G G G    
974	. . . . . . . . . . . . . . . . . T . T T T T T T    
975	. . . . . . . . . . . . . . . . . G . G G G G G G    
976	. . . . . . . . . . . . . . . . . A . A A A A A A    
977	. . . . . . . . . . . . . . . . . A . A A A A A A    
978	. . . . . . . . . . . . . . . . . G . G G G G G G    
979	. . . . . . . . . . . . . . . . . A . A T A A A A    
980	. . . . . . . . . . . . . . . . . . . G G G G G G    
981	. . . . . . . . . . . . . . . . . . . G G G G G G    
982	. . . . . . . . . . . . . . . . . . . G G G G G G    
983	. . . . . . . . . . . . . . . . . . . A A A A A A    
984	. . . . . . . . . . . . . . . . . . . G G G G G G    
985	. . . . . . . . . . . . . . . . . . . T T T T T T    
986	. . . . . . . . . . . . . . . . . . . A A A A A A    
987	. . . . . . . . . . . . . . . . . . . C C C C C C    
988	. . . . . . . . . . . . . . . . . . . A A A A A A    
989	. . . . . . . . . . . . . . . . . . . A A A A A A    
990	. . . . . . . . . . . . . . . . . . . C C C C C C    
991	. . . . . . . . . . . . . . . . . . . T T T T T T    
992	. . . . . . . . . . . . . . . . . . . G G G G G G    
993	. . . . . . . . . . . . . . . . . . . G G G G G G    
994	. . . . . . . . . . . . . . . . . . . C C C C C C    
995	. . . . . . . . . . . . . . . . . . . A A A A A A    
996	. . . . . . . . . . . . . . . . . . . A A A A A A    
997	. . . . . . . . . . . . . . . . . . . G G G G G G    
998	. . . . . . . . . . . . . . . . . . . T T T T T T    
999	. . . . . . . . . . . . . . . . . . . T T T T T T    
1000	. . . . . . . . . . . . . . . . . . . C C C C C C    
1001	. . . . . . . . . . . . . . . . . . . A A A A A A    
1002	. . . . . . . . . . . . . . . . . . . A A A A A A    
1003	. . . . . . . . . . . . . . . . . . . A A A A A A    
1004	. . . . . . . . . . . . . . . . . . . T T T T T T    
1005	. . . . . . . . . . . . . . . . . . . G G G G G G    
1006	. . . . . . . . . . . . . . . . . . . G G G G G G    
1007	. . . . . . . . . . . . . . . . . . . C C C C C C    
1008	. . . . . . . . . . . . . . . . . . . C C C C C C    
1009	. . . . . . . . . . . . . . . . . . . A A A A A A    
1010	. . . . . . . . . . . . . . . . . . . T T T T T T    
1011	. . . . . . . . . . . . . . . . . . . G G G G G G    
1012	. . . . . . . . . . . . . . . . . . . A A A A A A    
1013	. . . . . . . . . . . . . . . . . . . C C C C C C    
1014	. . . . . . . . . . . . . . . . . . . A A A A A A    
1015	. . . . . . . . . . . . . . . . . . . A A A A A A    
1016	. . . . . . . . . . . . . . . . . . . G G G G G G    
1017	. . . . . . . . . . . . . . . . . . . A A A A A A    
1018	. . . . . . . . . . . . . . . . . . . A A A A A A    
1019	. . . . . . . . . . . . . . . . . . . A A A A A A    
1020	. . . . . . . . . . . . . . . . . . . G G G G G G    
1021	. . . . . . . . . . . . . . . . . . . A A A A A A    
1022	. . . . . . . . . . . . . . . . . . . A A A A A A    
1023	. . . . . . . . . . . . . . . . . . . A A A A A A    
1024	. . . . . . . . . . . . . . . . . . . G G G G G G    
1025	. . . . . . . . . . . . . . . . . . . A A A A A A    
1026	. . . . . . . . . . . . . . . . . . . A A A A A A    
1027	. . . . . . . . . . . . . . . . . . . A A A A A A    
1028	. . . . . . . . . . . . . . . . . . . G G G G G G    
1029	. . . . . . . . . . . . . . . . . . . A A A A A A    
1030	. . . . . . . . . . . . . . . . . . . A A A A A A    
1031	. . . . . . . . . . . . . . . . . . . A A A A A A    
1032	. . . . . . . . . . . . . . . . . . . A A A A A A    
1033	. . . . . . . . . . . . . . . . . . . A A A A A A    
1034	. . . . . . . . . . . . . . . . . . . A A A A A A    
1035	. . . . . . . . . . . . . . . . . . . G G G G G G    
1036	. . . . . . . . . . . . . . . . . . . A . A A A A    
1037	. . . . . . . . . . . . . . . . . . . A . A A A A    
1038	. . . . . . . . . . . . . . . . . . . A . A A A A    
1039	. . . . . . . . . . . . . . . . . . . - . - - - G    
1040	. . . . . . . . . . . . . . . . . . . - . - - - A    
1041	. . . . . . . . . . . . . . . . . . . - . - - - A    
1042	. . . . . . . . . . . . . . . . . . . - . - - - A    
1043	. . . . . . . . . . . . . . . . . . . G . G G G G    
1044	. . . . . . . . . . . . . . . . . . . T . T T T T    
1045	. . . . . . . . . . . . . . . . . . . A . A A A A    
1046	. . . . . . . . . . . . . . . . . . . A . A A A A    
1047	. . . . . . . . . . . . . . . . . . . G . G G G G    
1048	. . . . . . . . . . . . . . . . . . . A . A A A A    
1049	. . . . . . . . . . . . . . . . . . . A . A A A A    
1050	. . . . . . . . . . . . . . . . . . . A . A A A C    
1051	. . . . . . . . . . . . . . . . . . . A . A A A A    
1052	. . . . . . . . . . . . . . . . . . . T . T T T T    
1053	. . . . . . . . . . . . . . . . . . . A . A A A A    
1054	. . . . . . . . . . . . . . . . . . . A . A A A A    
1055	. . . . . . . . . . . . . . . . . . . G . G G G G    
1056	. . . . . . . . . . . . . . . . . . . A . A A A A    
1057	. . . . . . . . . . . . . . . . . . . T . T T T G    
1058	. . . . . . . . . . . . . . . . . . . G . G G G G    
1059	. . . . . . . . . . . . . . . . . . . G . G G G G    
1060	. . . . . . . . . . . . . . . . . . . A . A A A A    
1061	. . . . . . . . . . . . . . . . . . . G . G G G G    
1062	. . . . . . . . . . . . . . . . . . . A . A A A A    
1063	. . . . . . . . . . . . . . . . . . . A . A A A A    
1064	. . . . . . . . . . . . . . . . . . . A . A A A A    
1065	. . . . . . . . . . . . . . . . . . . G . G G G G    
1066	. . . . . . . . . . . . . . . . . . . T . T T T T    
1067	. . . . . . . . . . . . . . . . . . . A . A A A A    
1068	. . . . . . . . . . . . . . . . . . . A . A A A A    
1069	. . . . . . . . . . . . . . . . . . . G . G G G G    
1070	. . . . . . . . . . . . . . . . . . . A . A A A A    
1071	. . . . . . . . . . . . . . . . . . . A . A A A A    
1072	. . . . . . . . . . . . . . . . . . . C . C C C C    
1073	. . . . . . . . . . . . . . . . . . . A . A A A A    
1074	. . . . . . . . . . . . . . . . . . . T . T T T T    
1075	. . . . . . . . . . . . . . . . . . . A . A A A A    
1076	. . . . . . . . . . . . . . . . . . . A . A A A A    
1077	. . . . . . . . . . . . . . . . . . . C . G G G G    
1078	. . . . . . . . . . . . . . . . . . . . . A A A A    
1079	. . . . . . . . . . . . . . . . . . . . . G G G G    
1080	. . . . . . . . . . . . . . . . . . . . . G G G G    
1081	. . . . . . . . . . . . . . . . . . . . . G G G G    
1082	. . . . . . . . . . . . . . . . . . . . . A A A A    
1083	. . . . . . . . . . . . . . . . . . . . . G G G G    
1084	. . . . . . . . . . . . . . . . . . . . . A A A A    
1085	. . . . . . . . . . . . . . . . . . . . . A A A A    
1086	. . . . . . . . . . . . . . . . . . . . . A A A A    
1087	. . . . . . . . . . . . . . . . . . . . . G G G G    
1088	. . . . . . . . . . . . . . . . . . . . . T T T T    
1089	. . . . . . . . . . . . . . . . . . . . . A A A A    
1090	. . . . . . . . . . . . . . . . . . . . . A A A A    
1091	. . . . . . . . . . . . . . . . . . . . . G G G G    
1092	. . . . . . . . . . . . . . . . . . . . . C A A A    
1093	. . . . . . . . . . . . . . . . . . . . . . A A A    
1094	. . . . . . . . . . . . . . . . . . . . . . C C C    
1095	. . . . . . . . . . . . . . . . . . . . . . A A A    
1096	. . . . . . . . . . . . . . . . . . . . . . T T T    
1097	. . . . . . . . . . . . . . . . . . . . . . A A A    
1098	. . . . . . . . . . . . . . . . . . . . . . A A A    
1099	. . . . . . . . . . . . . . . . . . . . . . G G G    
1100	. . . . . . . . . . . . . . . . . . . . . . A A A    
1101	. . . . . . . . . . . . . . . . . . . . . . G G G    
1102	. . . . . . . . . . . . . . . . . . . . . . G G G    
1103	. . . . . . . . . . . . . . . . . . . . . . G G G    
1104	. . . . . . . . . . . . . . . . . . . . . . A A A    
1105	. . . . . . . . . . . . . . . . . . . . . . G G G    
1106	. . . . . . . . . . . . . . . . . . . . . . A A A    
1107	. . . . . . . . . . . . . . . . . . . . . . A A A    
1108	. . . . . . . . . . . . . . . . . . . . . . A A A    
1109	. . . . . . . . . . . . . . . . . . . . . . G G G    
1110	. . . . . . . . . . . . . . . . . . . . . . T T T    
1111	. . . . . . . . . . . . . . . . . . . . . . A A A    
1112	. . . . . . . . . . . . . . . . . . . . . . A A A    
1113	. . . . . . . . . . . . . . . . . . . . . . G G G    
1114	. . . . . . . . . . . . . . . . . . . . . . A A A    
1115	. . . . . . . . . . . . . . . . . . . . . . A A A    
1116	. . . . . . . . . . . . . . . . . . . . . . C C C    
1117	. . . . . . . . . . . . . . . . . . . . . . A A A    
1118	. . . . . . . . . . . . . . . . . . . . . . T T T    
1119	. . . . . . . . . . . . . . . . . . . . . . A A A    
1120	. . . . . . . . . . . . . . . . . . . . . . A A A    
1121	. . . . . . . . . . . . . . . . . . . . . . G G G    
1122	. . . . . . . . . . . . . . . . . . . . . . A A A    
1123	. . . . . . . . . . . . . . . . . . . . . . T G G    
1124	. . . . . . . . . . . . . . . . . . . . . . . . G    
1125	. . . . . . . . . . . . . . . . . . . . . . . . G    
1126	. . . . . . . . . . . . . . . . . . . . . . . . A    
1127	. . . . . . . . . . . . . . . . . . . . . . . . G    
1128	. . . . . . . . . . . . . . . . . . . . . . . . A    
1129	. . . . . . . . . . . . . . . . . . . . . . . . A    
1130	. . . . . . . . . . . . . . . . . . . . . . . . A    
1131	. . . . . . . . . . . . . . . . . . . . . . . . G    
1132	. . . . . . . . . . . . . . . . . . . . . . . . T    
1133	. . . . . . . . . . . . . . . . . . . . . . . . A    
1134	. . . . . . . . . . . . . . . . . . . . . . . . A    
1135	. . . . . . . . . . . . . . . . . . . . . . . . G    
1136	. . . . . . . . . . . . . . . . . . . . . . . . A    
1137	. . . . . . . . . . . . . . . . . . . . . . . . A    
1138	. . . . . . . . . . . . . . . . . . . . . . . . C    
1139	. . . . . . . . . . . . . . . . . . . . . . . . A    
1140	. . . . . . . . . . . . . . . . . . . . . . . . T    
1141	. . . . . . . . . . . . . . . . . . . . . . . . A    
1142	. . . . . . . . . . . . . . . . . . . . . . . . A    
1143	. . . . . . . . . . . . . . . . . . . . . . . . G    
1144	. . . . . . . . . . . . . . . . . . . . . . . . A    
1145	. . . . . . . . . . . . . . . . . . . . . . . . G    
1146	. . . . . . . . . . . . . . . . . . . . . . . . G    
1147	. . . . . . . . . . . . . . . . . . . . . . . . G    
1148	. . . . . . . . . . . . . . . . . . . . . . . . A    
1149	. . . . . . . . . . . . . . . . . . . . . . . . G    
1150	. . . . . . . . . . . . . . . . . . . . . . . . A    
1151	. . . . . . . . . . . . . . . . . . . . . . . . A    
1152	. . . . . . . . . . . . . . . . . . . . . . . . A    
1153	. . . . . . . . . . . . . . . . . . . . . . . . G    
1154	. . . . . . . . . . . . . . . . . . . . . . . . T    
1155	. . . . . . . . . . . . . . . . . . . . . . . . A    
1156	. . . . . . . . . . . . . . . . . . . . . . . . A    
1157	. . . . . . . . . . . . . . . . . . . . . . . . G    
1158	. . . . . . . . . . . . . . . . . . . . . . . . A    
1159	. . . . . . . . . . . . . . . . . . . . . . . . A    
1160	. . . . . . . . . . . . . . . . . . . . . . . . A    
1161	. . . . . . . . . . . . . . . . . . . . . . . . A    
1162	. . . . . . . . . . . . . . . . . . . . . . . . T    
1163	. . . . . . . . . . . . . . . . . . . . . . . . A    
1164	. . . . . . . . . . . . . . . . . . . . . . . . A    
1165	. . . . . . . . . . . . . . . . . . . . . . . . G    
1166	. . . . . . . . . . . . . . . . . . . . . . . . A    
1167	. . . . . . . . . . . . . . . . . . . . . . . . G    
1168	. . . . . . . . . . . . . . . . . . . . . . . . G    
1169	. . . . . . . . . . . . . . . . . . . . . . . . G    
1170	. . . . . . . . . . . . . . . . . . . . . . . . A    
1171	. . . . . . . . . . . . . . . . . . . . . . . . G    
1172	. . . . . . . . . . . . . . . . . . . . . . . . A    
1173	. . . . . . . . . . . . . . . . . . . . . . . . A    
1174	. . . . . . . . . . . . . . . . . . . . . . . . A    
1175	. . . . . . . . . . . . . . . . . . . . . . . . G    
1176	. . . . . . . . . . . . . . . . . . . . . . . . T    
1177	. . . . . . . . . . . . . . . . . . . . . . . . A    
1178	. . . . . . . . . . . . . . . . . . . . . . . . A    
1179	. . . . . . . . . . . . . . . . . . . . . . . . G    
1180	. . . . . . . . . . . . . . . . . . . . . . . . A    
1181	. . . . . . . . . . . . . . . . . . . . . . . . A    
1182	. . . . . . . . . . . . . . . . . . . . . . . . C    
1183	. . . . . . . . . . . . . . . . . . . . . . . . A    
1184	. . . . . . . . . . . . . . . . . . . . . . . . T    
1185	. . . . . . . . . . . . . . . . . . . . . . . . A    
1186	. . . . . . . . . . . . . . . . . . . . . . . . A    
1187	. . . . . . . . . . . . . . . . . . . . . . . . G    
1188	. . . . . . . . . . . . . . . . . . . . . . . . A    
1189	. . . . . . . . . . . . . . . . . . . . . . . . G    
1190	. . . . . . . . . . . . . . . . . . . . . . . . G    
1191	. . . . . . . . . . . . . . . . . . . . . . . . G    
1192	. . . . . . . . . . . . . . . . . . . . . . . . A    
1193	. . . . . . . . . . . . . . . . . . . . . . . . G    
1194	. . . . . . . . . . . . . . . . . . . . . . . . A    
1195	. . . . . . . . . . . . . . . . . . . . . . . . A    
1196	. . . . . . . . . . . . . . . . . . . . . . . . A    
1197	. . . . . . . . . . . . . . . . . . . . . . . . G    
1198	. . . . . . . . . . . . . . . . . . . . . . . . T    
1199	. . . . . . . . . . . . . . . . . . . . . . . . A    
1200	. . . . . . . . . . . . . . . . . . . . . . . . A    
1201	. . . . . . . . . . . . . . . . . . . . . . . . G    
1202	. . . . . . . . . . . . . . . . . . . . . . . . A    
1203	. . . . . . . . . . . . . . . . . . . . . . . . A    
1204	. . . . . . . . . . . . . . . . . . . . . . . . C    
1205	. . . . . . . . . . . . . . . . . . . . . . . . A    
1206	. . . . . . . . . . . . . . . . . . . . . . . . T    
1207	. . . . . . . . . . . . . . . . . . . . . . . . A    
1208	. . . . . . . . . . . . . . . . . . . . . . . . A    
1209	. . . . . . . . . . . . . . . . . . . . . . . . G    
1210	. . . . . . . . . . . . . . . . . . . . . . . . A    
1211	. . . . . . . . . . . . . . . . . . . . . . . . G    
1212	. . . . . . . . . . . . . . . . . . . . . . . . G    
1213	. . . . . . . . . . . . . . . . . . . . . . . . G    
1214	. . . . . . . . . . . . . . . . . . . . . . . . A    
1215	. . . . . . . . . . . . . . . . . . . . . . . . G    
1216	. . . . . . . . . . . . . . . . . . . . . . . . A    
1217	. . . . . . . . . . . . . . . . . . . . . . . . A    
1218	. . . . . . . . . . . . . . . . . . . . . . . . A    
1219	. . . . . . . . . . . . . . . . . . . . . . . . G    
1220	. . . . . . . . . . . . . . . . . . . . . . . . T    
1221	. . . . . . . . . . . . . . . . . . . . . . . . A    
1222	. . . . . . . . . . . . . . . . . . . . . . . . A    
1223	. . . . . . . . . . . . . . . . . . . . . . . . G    
1224	. . . . . . . . . . . . . . . . . . . . . . . . A    
1225	. . . . . . . . . . . . . . . . . . . . . . . . A    
1226	. . . . . . . . . . . . . . . . . . . . . . . . C    
1227	. . . . . . . . . . . . . . . . . . . . . . . . A    
1228	. . . . . . . . . . . . . . . . . . . . . . . . T    
1229	. . . . . . . . . . . . . . . . . . . . . . . . A    
1230	. . . . . . . . . . . . . . . . . . . . . . . . A    
1231	. . . . . . . . . . . . . . . . . . . . . . . . G    
1232	. . . . . . . . . . . . . . . . . . . . . . . . A    
1233	. . . . . . . . . . . . . . . . . . . . . . . . G    
1234	. . . . . . . . . . . . . . . . . . . . . . . . G    
1235	. . . . . . . . . . . . . . . . . . . . . . . . G    
1236	. . . . . . . . . . . . . . . . . . . . . . . . A    
1237	. . . . . . . . . . . . . . . . . . . . . . . . G    
1238	. . . . . . . . . . . . . . . . . . . . . . . . A    
1239	. . . . . . . . . . . . . . . . . . . . . . . . A    
1240	. . . . . . . . . . . . . . . . . . . . . . . . A    
1241	. . . . . . . . . . . . . . . . . . . . . . . . G    
1242	. . . . . . . . . . . . . . . . . . . . . . . . T    
1243	. . . . . . . . . . . . . . . . . . . . . . . . A    
1244	. . . . . . . . . . . . . . . . . . . . . . . . A    
1245	. . . . . . . . . . . . . . . . . . . . . . . . G    
1246	. . . . . . . . . . . . . . . . . . . . . . . . A    
1247	. . . . . . . . . . . . . . . . . . . . . . . . A    
1248	. . . . . . . . . . . . . . . . . . . . . . . . C    
1249	. . . . . . . . . . . . . . . . . . . . . . . . A    
1250	. . . . . . . . . . . . . . . . . . . . . . . . T    
1251	. . . . . . . . . . . . . . . . . . . . . . . . A    
1252	. . . . . . . . . . . . . . . . . . . . . . . . A    
1253	. . . . . . . . . . . . . . . . . . . . . . . . G    
1254	. . . . . . . . . . . . . . . . . . . . . . . . A    
1255	. . . . . . . . . . . . . . . . . . . . . . . . G    
1256	. . . . . . . . . . . . . . . . . . . . . . . . G    
1257	. . . . . . . . . . . . . . . . . . . . . . . . G    
1258	. . . . . . . . . . . . . . . . . . . . . . . . A    
1259	. . . . . . . . . . . . . . . . . . . . . . . . G    
1260	. . . . . . . . . . . . . . . . . . . . . . . . A    
1261	. . . . . . . . . . . . . . . . . . . . . . . . A    
1262	. . . . . . . . . . . . . . . . . . . . . . . . A    
1263	. . . . . . . . . . . . . . . . . . . . . . . . G    
1264	. . . . . . . . . . . . . . . . . . . . . . . . T    
1265	. . . . . . . . . . . . . . . . . . . . . . . . A    
1266	. . . . . . . . . . . . . . . . . . . . . . . . A    
1267	. . . . . . . . . . . . . . . . . . . . . . . . G    
1268	. . . . . . . . . . . . . . . . . . . . . . . . A    
1269	. . . . . . . . . . . . . . . . . . . . . . . . A    
1270	. . . . . . . . . . . . . . . . . . . . . . . . C    
1271	. . . . . . . . . . . . . . . . . . . . . . . . A    
1272	. . . . . . . . . . . . . . . . . . . . . . . . T    
1273	. . . . . . . . . . . . . . . . . . . . . . . . A    
1274	. . . . . . . . . . . . . . . . . . . . . . . . A    
1275	. . . . . . . . . . . . . . . . . . . . . . . . G    
1276	. . . . . . . . . . . . . . . . . . . . . . . . A    
1277	. . . . . . . . . . . . . . . . . . . . . . . . G    
1278	. . . . . . . . . . . . . . . . . . . . . . . . G    
1279	. . . . . . . . . . . . . . . . . . . . . . . . G    
1280	. . . . . . . . . . . . . . . . . . . . . . . . A    
1281	. . . . . . . . . . . . . . . . . . . . . . . . G    
1282	. . . . . . . . . . . . . . . . . . . . . . . . A    
1283	. . . . . . . . . . . . . . . . . . . . . . . . A    
1284	. . . . . . . . . . . . . . . . . . . . . . . . A    
1285	. . . . . . . . . . . . . . . . . . . . . . . . G    
1286	. . . . . . . . . . . . . . . . . . . . . . . . T    
1287	. . . . . . . . . . . . . . . . . . . . . . . . A    
1288	. . . . . . . . . . . . . . . . . . . . . . . . A    
1289	. . . . . . . . . . . . . . . . . . . . . . . . G    
1290	. . . . . . . . . . . . . . . . . . . . . . . . A    
1291	. . . . . . . . . . . . . . . . . . . . . . . . A    
1292	. . . . . . . . . . . . . . . . . . . . . . . . A    
1293	. . . . . . . . . . . . . . . . . . . . . . . . G    
1294	. . . . . . . . . . . . . . . . . . . . . . . . A    
1295	. . . . . . . . . . . . . . . . . . . . . . . . G    
1296	. . . . . . . . . . . . . . . . . . . . . . . . G    
1297	. . . . . . . . . . . . . . . . . . . . . . . . G    
1298	. . . . . . . . . . . . . . . . . . . . . . . . A    
1299	. . . . . . . . . . . . . . . . . . . . . . . . A    
1300	. . . . . . . . . . . . . . . . . . . . . . . . G    
1301	. . . . . . . . . . . . . . . . . . . . . . . . A    
1302	. . . . . . . . . . . . . . . . . . . . . . . . C    
1303	. . . . . . . . . . . . . . . . . . . . . . . . T    
1304	. . . . . . . . . . . . . . . . . . . . . . . . T    
1305	. . . . . . . . . . . . . . . . . . . . . . . . A    
1306	. . . . . . . . . . . . . . . . . . . . . . . . A    
1307	. . . . . . . . . . . . . . . . . . . . . . . . C    
1308	. . . . . . . . . . . . . . . . . . . . . . . . A    
1309	. . . . . . . . . . . . . . . . . . . . . . . . G    
1310	. . . . . . . . . . . . . . . . . . . . . . . . C    
1311	. . . . . . . . . . . . . . . . . . . . . . . . C    
1312	. . . . . . . . . . . . . . . . . . . . . . . . C
```
